# Nanoparticulate Copper Cluster-Mediated Biosensing of Cardiac Biomolecular Markers

**DOI:** 10.3390/bios15040237

**Published:** 2025-04-08

**Authors:** Lakshmi V. Nair, Jarred Wheeler, Yaelyn Ha, Kimberly M. Jones, Jesse Jones, Vinoy Thomas

**Affiliations:** 1 Department of Mechanical and Materials Engineering, School of Engineering, University of Alabama at Birmingham, Birmingham, AL 35294, USA; lvijayan@uab.edu (L.V.N.); jwheele@uab.edu (J.W.); haily07@uab.edu (Y.H.); brianna@uab.edu (K.M.J.); 2 Department of Neurosurgery, School of Medicine, University of Alabama at Birmingham, Birmingham, AL 35294, USA; jessejones@uabmc.edu; 3 Center for Nanoscale Materials and Bio-Integration (CNMB), University of Alabama at Birmingham, Birmingham, AL 35294, USA; 4 Center for Clinical and Translational Sciences (CCTS), University of Alabama at Birmingham, Birmingham, AL 35294, USA

**Keywords:** biosensor, copper, troponin, cardiac biomarker

## Abstract

Being a leading cause of death, heart diseases across the globe need special attention to enable early diagnosis. Metal nanoparticle-mediated biosensors are useful clinical tools for the early detection of bio-analytes. The size-dependent surface plasmon resonance (SPR) of metal nanoparticles can be effectively utilized for the same purpose. The early detection of heart diseases can be evaluated by monitoring the troponin level. A copper nanoparticle-mediated troponin biosensor was developed through antibody conjugation for troponin I and troponin T. The copper nanosensor shows a concentration-dependent SPR change towards troponin T and troponin I.

## 1. Introduction

Despite the vast advancements in the progress of science and technology, heart-related diseases remain among the leading causes of death [1,2]. They kill hundreds of thousands of individuals each year, and this trend does not seem to be slowing down. According to the 2019 report by the American Heart Association, 48 percent of adults in the US have cardiovascular diseases. Effective and early diagnosis is the best method to address these diseases. The early detection of cardiovascular diseases could help to reduce these numbers. Troponin is one of the markers used for the determination of heart-related diseases [3,4,5,6]. Troponin falls under the category of cardiac markers. Cardiac markers are the substances working in our bloodstream as indicators of injured muscles in the heart. They can be used to examine and follow up myocardial infarction as damaged heart muscles increase the release of these markers. Measuring blood samples to indicate differences in levels of these markers can help cardiac marker sensors, especially in detecting the damage signals. Specifically, troponin I, specifically Cardiac troponin T (cTnT), is a suitable protein for use in biosensors due to its characteristic of surviving more than creatinine kinase-MB based on statistics from the American Heart Association (AHA).

This allows for hospitals to check for the risk of cardiovascular diseases, as well as the severity of heart attacks. There are two main types of troponin proteins: troponin I and troponin T. The amount of troponin proteins is checked against a reference amount determined to be 0–0.04 ng/mL of troponin I and 0–0.01 ng/mL of troponin T. Troponin at higher concentrations indicates that the level is higher than that in 99% of healthy adults. Developing a cost-effective, reliable and effective sensor for both troponin T and troponin I is an effective method to evaluate troponin and related risk levels.

Nanomaterial-enabled biosensors are one of the effective approaches for the early-stage detection of biomarkers with high selectivity and efficacy. Noble metal nanoparticles, quantum dots, and carbon nanomaterial-based sensors are of great importance for the quantification of analytes of interest using their inherent optical properties [7,8,9,10,11,12,13,14]. These materials exhibit size-dependent optical properties from the visible to near infra-red (NIR) regions of the electromagnetic spectrum. Among the different nanomaterials, metallic nanomaterials are useful for their size-dependent surface plasmon resonance properties. Among the metal nanomaterials, the noble metal nanomaterials gold (Au) and silver (Ag) have received widespread attention due to their size tunable surface plasmon resonance behaviors. The sensitivity and selectivity of these metal nanoparticles are very high, and as a result, they are used as effective sensors in diagnostic and therapeutic applications. For example, gold nanoparticles can be used to create electrochemical sensors based on their high-conductivity immobilizing antibodies related to troponin proteins. When troponin binds to the nanoparticles, sensors can detect and analyze the signals to identify the functionalization of the sensor and, hence, enhance signal detections [15,16,17,18]. More specifically, in the scope of early detection, they can also be used for antimicrobial therapy and cardio protection as gold nanoparticles have stable and target-specific characteristics compared to other materials, contributing to the controlled use of particles in leveraging specific conditions.

Nanomaterial-enabled cardiac sensors are one of the important area research areas for detecting elevated troponin in the body as an early indicator for myocardial infraction [19,20,21]. Bhatnagar et al. developed a troponin I-conjugated nanohybrid sensor made of graphene quantum dot for the detection of heart attack [22]. A cardiac troponin T sensor was developed using a label-free aptamer-based field-effect transistor using gold nanoparticles, metal oxide nanorods and single-wall carbon nanotubes [23]. Different nanomaterial sensors were used for the detection of troponin via a troponin sensor [24,25,26]. Compared to Au and Ag, copper (Cu) is the 25th most abundant metal in the Earth’s crust, which makes it a cost-effective option. Due to the abundant and inexpensive nature of copper, Cu-based nanomaterials have been of great interest in recent years, especially in the areas of catalysis and biomedicine [27]. Copper nanoparticles are also known to be one of the excellent engineered materials for biosensing applications because of their noticeably high optical, thermal, and electrical properties. The highly conductive background of copper helps to transfer electrons and biomolecules like troponin and other enzymatic substances, which contributes to the higher sensitivity of a device [28,29]. Copper also exhibits antimicrobial properties, making it suitable in cellular backgrounds. Using biocompatible materials lead the device to be rather functionalized and dedicated to its therapeutic purposes with fewer concerns about side effects. Moreover, the plasmonic Cu nanomaterial-based biosensors show superior sensitivity due to their strong optical properties. Their highly absorbing and scattering nature helps copper nanoparticles to become stabilizers and maintain plasmonic properties [30]. Thus, copper-based nanomaterials, after suitable modifications, can be used as biosensors for glucose and Micro-RNA153 [31,32,33,34,35]. Most of the approaches used are based on electrochemical and immunosensor methods [36,37]. Herein, we report a Cu nanoparticle-based biosensor with NIR absorbance for the detection of biomolecules such as troponin I and troponin T. The NIR absorption of the material is highly advantageous for avoiding autofluorescence interference by biofluids such as blood.

## 2. Materials and Methods

### 2.1. Chemicals

All the chemicals used in this experiment were used as such unless stated otherwise. The chemicals used were Cetyltrimethylammonium Bromide (CTAB) obtained from MP Biomedicals, Copper (II) sulfate pentahydrate 99%, and Sodium borohydride 98% obtained from Fischer scientific. Cardiac Troponin T recombinant rabbit monoclonal antibody (17H8L13) and Cardiac Troponin I monoclonal antibody (16A11) were obtained using Invitrogen by Thermo Fisher Scientific. Recombinant cardiac troponin I and recombinant cardiac T troponin protein were obtained from Abcam.

### 2.2. Methods

The surface plasmon resonances of the synthesized copper nanoparticles and antibody-conjugated copper nanoparticles and their sensing efficacies were recorded using the Perkin Elmer UV-Vis-NIR double-beam absorbance spectrophotometer (LAMBDA 1050) by dispersal in solvent. The spectra were recorded using a 1 cm path length quartz cuvette.

The chemical state and elemental composition of the copper nanoparticles was confirmed using X-ray photoelectron spectroscopy (XPS). The XPS spectra were recorded using an X-ray photoelectron spectroscope (phi 5000). The copper nanoparticles were lyophilized, and the powdered samples were used for the identification of the compound. Both the survey spectrum and high-resolution spectra were recorded for the analysis of the synthesized copper nanoparticles.

The functional group present on the copper nanoparticles was confirmed using Fourier transform infra-red spectroscopy (FTIR). FTIR were recorded using a Bruker FTIR spectrometer in ATR mode with 120 scans, with a wavenumber ranging from 4000 cm^−1^ to 400 cm^−1^.

The morphologies of the copper nanoparticles before and after antibody conjugation were confirmed using transmission electron microscopy. The mechanistic actions of antigens upon interaction with the corresponding antibody-conjugated copper nanoparticles were further confirmed using TEM analysis. TEM results were recorded using a JEOL 1400 TEM instrument equipped with an AMT-NanoSprint43L-MarkII Camera. The copper nanoparticles, troponin I-conjugated CuNP, troponin T-conjugated CuNP, and their antigen-treated nanosensors were drop-casted onto a carbon-treated TEM grid with an accelerating voltage of 120 kV.

### 2.3. Synthesis of Copper Nanoparticles (CuNPs)

The wet chemical method was used for the synthesis of copper nanoparticles using copper sulphate and reduced glutathione. Sodium borohydride was used as a reducing agent for synthesizing copper nanoparticles (CuNPs). In brief, 20 mg of sodium borohydride powder was dissolved in 10 mL of deionized water (5.29 × 10^−4^ mol) kept in a refrigerator. Next, 2 mg of CTAB dissolved in 3 mL of deionized water (5.22 × 10^−6^ mol) was treated with 6.2 mg of copper sulfate in 1 mL water (3.89 × 10^−5^ mol). It was critical to ensure that copper added to CTAB was fully dissolved without any bubble formation. The mixture was allowed to be stirred for another 15 min with a total volume of 5 mL of deionized water. After 15 min, the sodium borohydride was added dropwise until the solution turned light brown. For the color to reach its optimum level, 75–100 µL was required. The reaction was allowed to continue for another one hour for the synthesis of CuNP. The synthesized copper nanoparticles were purified using centuriation at 15,000 rpm for 30 min. The surface plasmon resonance of the samples was recorded using a UV–visible absorbance spectrophotometer.

### 2.4. Synthesis of Troponin I Monoclonal Antibody-Conjugated Copper Nanoparticles

In total, 200 µg (5.9 mg/mL) of cardiac troponin monoclonal antibodies was dispersed in 200 µL of 1× phosphate-buffered saline (PBS). A working solution of cardiac troponin monoclonal antibody solution was prepared by mixing 30 µL of the stock solution with 1 mL of 1× phosphate-buffered saline (PBS). From this, 100 µL of working solution of cardiac troponin I antibody was added to the copper nanoparticle solution and stirred for three hours. The synthesized cardiac troponin monoclonal antibody-conjugated copper nanoparticles were purified using centrifugation at 15,000 rpm for 30 min.

### 2.5. Synthesis of Troponin T Monoclonal Antibody-Conjugated Copper Nanoparticles

A total of 100 µg of cardiac troponin T recombinant rabbit monoclonal antibody (0.5 mg/mL) was used for this study. A working solution of the troponin T monoclonal antibody was prepared by mixing 100 µL of the antibody in 1 mL of PBS. A total of 100 µL of the troponin T monoclonal antibody from the working solution was added to 1 mL of the copper nanoparticles dropwise and stirred for another 3 h. After 3 h, troponin T-conjugated copper nanoparticles were purified by centrifugation at 15,000 rpm for 30 min.

### 2.6. Detection of Troponin I and T by the Troponin I and Tmonoclonal Antibody-Conjugated Copper Nanoparticles

The purified troponin I-conjugated copper nanoparticles were dispersed in 3 mL of PBS and added to different concentrations of human cardiac troponin I (0.25 mg/mL). The sensing efficacy of the developed sensor upon interaction with troponin I was recorded by monitoring the change in the surface plasmon resonance of the developed sensor using a UV–visible absorbance spectrophotometer.

Similarly, purified troponin T detected by troponin T monoclonal antibody-conjugated copper nanoparticles was dispersed in 3 mL of PBS and added to different concentration of human cardiac troponin T (0.25 mg/mL). The changes in the surface plasmon resonance of troponin T antibody-conjugated CuNP were monitored for the detection of troponin T antigen. A UV–visible absorbance spectrophotometer is used for the task.

## 3. Results

### 3.1. Synthesis and Characterization of Copper Nanoparticles

Copper nanoparticles were synthesized using the wet chemical method using copper sulphate pentahydrate and cetyltrimethylammonium bromide and sodium borohydride as the reducing agents. The color of the solution changed from colorless to brown after the addition of borohydride. The synthesized copper nanoparticle (CuNP) showed a surface plasmon resonance at around 800 nm, which was advantageous for a nanosensor to avoid light interference with body fluids.

**Figure 1:**
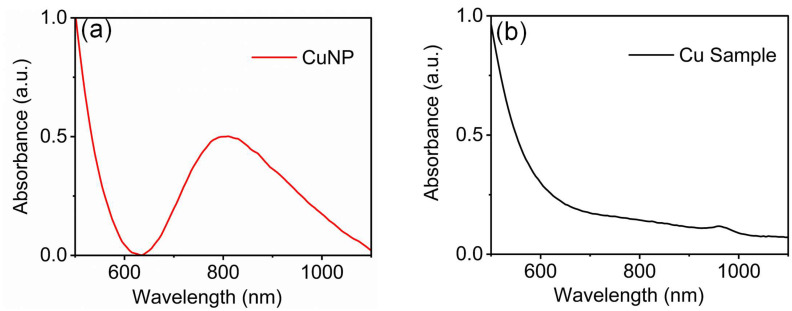
UV–visible absorbance spectra of (**a**) CuNP nanoparticles with freshly prepared sodium borohydride and (**b**) copper samples using older borohydride. CuNP was synthesized using the freshly prepared sodium borohydride shows surface plasmon resonance around 800 nm compared to Figure (**b**).

It should be noted that freshly prepared borohydride is necessary for the reaction. It is found that as the borohydride becomes older (around 32 to 48 h), it needs to increase the volume of addition and sometimes the reaction mixture becomes cloggy. This may be because sodium borohydride breaks down in neutral and acidic solutions NaBH_4_ + 2H_2_O = NaBO_2_ + 4H_2_. Once sodium borohydride is added to the copper and CTAB solution, it forms a brown/amber color solution and continues to mix for another 1 h. Figure 1 shows the UV–visible absorbance spectra of synthesized CuNP. The absorbance spectra of CuNP show a surface plasmon at around 800 nm, but as the sodium borohydride becomes older, the surface plasmon resonance disappears (Figure 1b).

**Figure 2:**
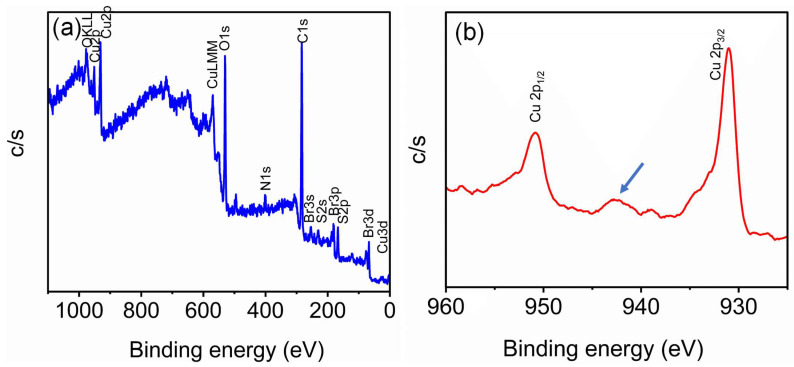
XPS spectra of CuNP: (**a**) survey spectra; (**b**) Cu high-resolution spectra. The survey spectra show that the binding energy corresponds to Cu, Br, N, and O. The high-resolution spectra of Cu show that the binding energy corresponds to Cu 2P_1/2_, Cu 2P_3/2_, and the satellite peak (arrow mark) at around 942.5 eV.

Furthermore, the elemental composition and chemical structure of the developed CuNP were characterized using X-ray photon correlation spectroscopy (XPS) and Fourier transform infra-red spectroscopy (FTIR). For XPS analysis, the lyophilized powders of the CuNP were used. Figure 2a shows the survey spectrum of the synthesized copper nanoparticles. In the survey spectrum, Cu 2p_3/2_ and Cu 2p_1/2_ indicate the presence of copper, along with oxygen, nitrogen, carbon, and bromine (Figure 2a). The origins of nitrogen and bromine are attributed to the presence of a CTAB molecule providing stability to the CuNP. From the high-resolution XPS spectra, the binding energies at around 931 eV and 951.1 eV confirm Cu 2p_3/2_ and Cu 2p_1/2_, respectively. Along with these peaks, the presence of a satellite peak at around 942.5 eV is observed in the high-resolution XPS spectra (Figure 2b). This indicates that synthesized CuNP oxidation is Cu^2+^ (Figure 2b). We expect a protective layer of copper oxide over the CuNP for providing additional stability to the synthesized nanoparticle.

**Figure 3:**
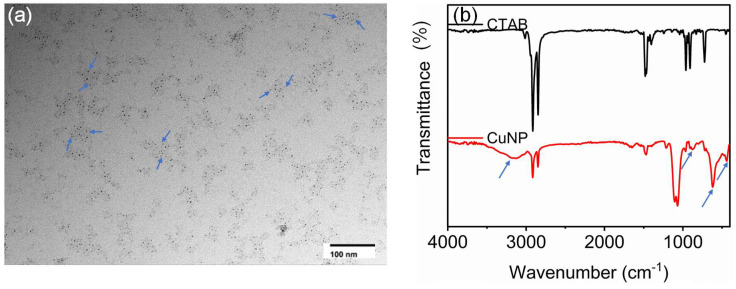
(**a**) TEM images of the CuNP and (**b**) FTIR spectra of CuNP. TEM image shows that a few 4 nm sized nanoparticles were encapsulated inside the transparent layer. The transparent layer is shown with arrows (blue) in Figure 3a. The scale bar is 100 nm. The FT IR spectra show a broader OH peak at 3145.45 cm^−1^, indicating the presence of bound water or a hydride layer. The arrow at 621 cm^−1^ shows the characteristic vibrations of Cu-O vibrations of CuO in CuNP.

These findings were further confirmed using the transmission electron microscopic images (Figure 3). From the TEM analysis, it is evident that a few 4 nm sized nanoparticles were encapsulated inside the transparent layer (Figure 3a). This transparent layer will give additional stability, thereby hindering further oxidation. This will provide excellent stability to the nanosensor and allow for further modification of different ligands and analytes. The nanosensor comprises a copper cluster protected with a layer of copper oxide.

The functional groups over the synthesized copper nanoparticles were further monitored using Fourier transform infra-red spectroscopy. From the FTIR spectra, a broader OH peak at 3145.45 cm^−1^ is observed. This indicates the presence of bound water or a hydride layer on the synthesized nanoparticles. The FTIR spectra also show vibrational bands at around 873 cm^−1^, 621 cm^−1^, and 440 cm^−1^, which is attributed to the characteristic vibrations of Cu-O in the CuNP [38,39,40]. The other characteristic vibrations are similar to that of CTAB, with a slight shift in their vibrational modes. The shift can be attributed to the binding with Cu (Figure 3b).

### 3.2. Development of CuNP Sensor for Troponin I and Troponin T

**Figure 4:**
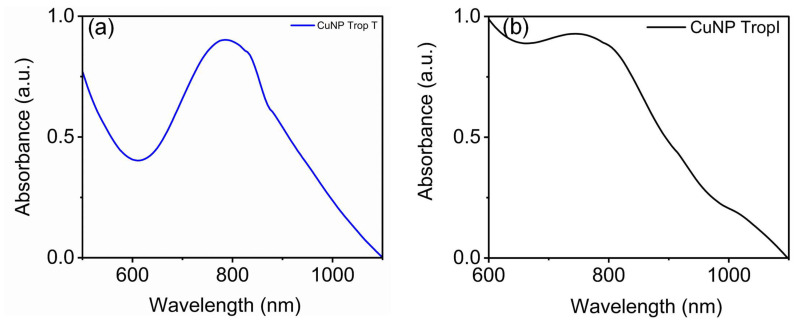
UV–visible absorbance spectra of (**a**) the troponin T antibody and (**b**) troponin I antibody-conjugated CuNP. The surface plasmon resonance of CuNP remains the same after functionalizing with the troponin T antibody, whereas troponin I antibody conjugation results in broadening.

For sensor development, corresponding antibodies specific for troponin I and troponin T were functionalized with the CuNP. In brief, a stock solution of troponin I antibody was prepared by mixing 200 µL the antibody in 5.9 mg/mL of cardiac troponin monoclonal antibody with 30 µL of the stock solution and combining it with 1 mL of 1× phosphate-buffered saline (PBS) used for the functionalization of CuNP. From this, 100 µL of working solution of troponin I antibody was added to the copper nanoparticle solution and stirred for three hours. After 3 h, troponin I antibody-conjugated CuNP nanoparticles were purified using centrifugation. For the fabrication of troponin T antibody-conjugated CuNP, a similar approach was established. These two nanosensors were separately tested for the detection of troponin I and troponin T. Enzyme-linked immunosorbent assay (ELISA), chemiluminescence, fluorescence analysis, and high-sensitivity cardiac troponin (hs-cTn) are the most commonly used methods for the detection cardiac troponin. These techniques require more time to detect the analytes, false negative results, and expensive chemicals. Figure 4 shows the surface plasmon properties of CuNP after binding with antibodies specific for troponin I and T.

In Figure 4a, troponin T antibody conjugation does not affect the nature of the SPR of copper nanoparticle. But, at the same time, antibody conjugation results in the slight blue shift of CuNP from 809 nm to 794 nm. Similarly, the conjugation of troponin I antibody with copper nanomaterial results in the broadening of the SPR. The appearance of the surface plasmon resonance of copper nanomaterial is different for both troponin I antibody and troponin T antibody conjugation. This is useful for identifying individual analytes such as troponin I and troponin T specifically with accuracy.

**Figure 5:**
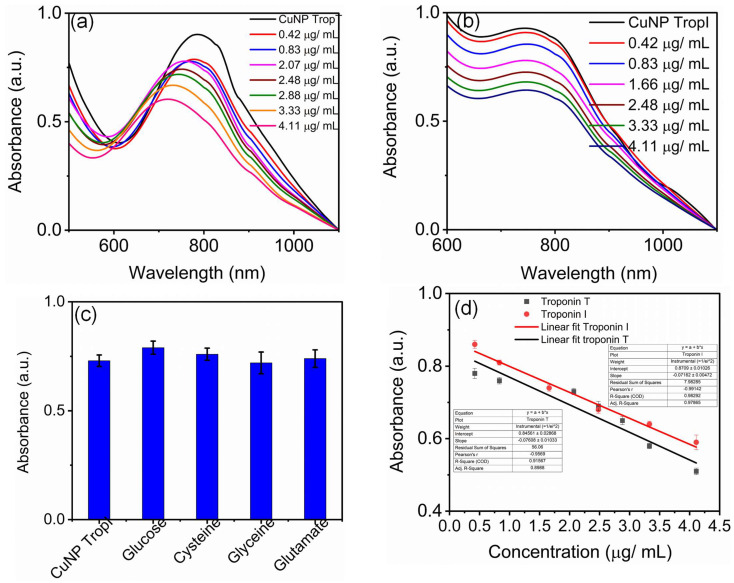
The surface plasmon resonance of (**a**) troponin T antibody-conjugated CuNP upon adding different concentrations of troponin T; (**b**) the surface plasmon resonance of troponin I antibody-conjugated CuNP upon adding different concentrations of troponin I. Antigen addition resulted in the quenching of the surface plasmon resonance of the respective antibody-conjugated CuNPs. (**c**) The selectivity of the developed sensor towards different analyte absorbances measured at 794 nm and (**d**) a plot showing the change in surface plasmon resonance upon adding different concentration of analytes with a linear fit.

Furthermore, the sensing efficacies of these antibody-conjugated nanosensors were monitored by adding known concentrations of troponin T and troponin I to the respective sensors. Figure 5 shows the change in the surface plasmon resonance upon adding the antigens. The sensitivity of the sensor is an important parameter that must be addressed when considered as a sensor. It should be noted that we used 0.25 mg/mL of troponin T and troponin I with a concentration ranging from 0.42 µg/mL to 4 µg/mL, with maximum volume of 50 µL, to avoid the dilution effect. Figure 5d shows the concentration-dependent change in the surface plasmon resonance of antibody-conjugated CuNP with a linear fit. The limits of detection of troponin I and troponin T were found to be 0.197 µg/mL and 0.419 µg/mL, respectively.

In Figure 5, troponin T antigen shows a concentration-dependent decrease in the SPR with a blue shift in the surface plasmon resonance. Troponin I antibody-conjugated CuNP shows a concentration-dependent decrease in the SPR. These changes in the SPR will help to quantify the antigen (troponin I and troponin T) present in the sample. The present study is a more qualitative approach for showing the efficacy of a copper nanocluster protected with a protective coating that can be used as a selective sensor for the detection of analytes of interest upon suitable functionalization and modification, as well as its mechanistic action.

**Figure 6:**
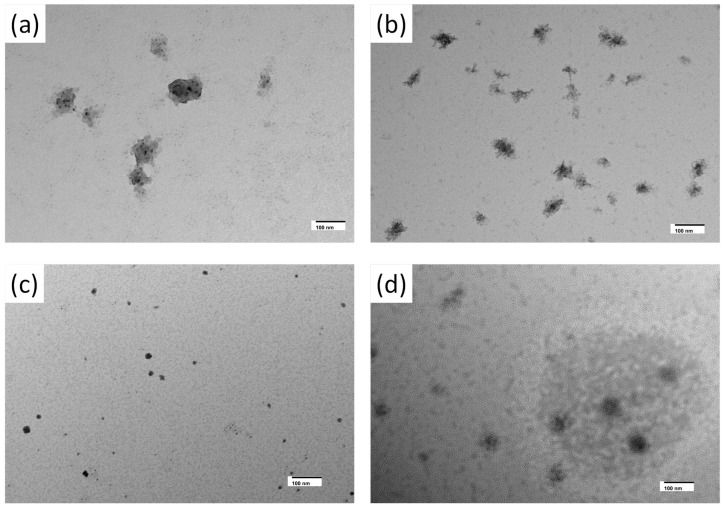
TEM images of (**a**) troponin T antibody-conjugated CuNP, (**b**) troponin T antibody-conjugated CuNP with a higher concentration of troponin T antigen, (**c**) troponin I antibody-conjugated CuNP, and (**d**) troponin I antibody-conjugated CuNP with a higher concentration of troponin I antigen. The change in the morphology of CuNP can be clearly observed in the TEM images.

The mechanisms of action of CuNP towards troponin I and troponin T were evident in the TEM images before and after adding the respective antigens. For the TEM, troponin T antibody binding did not alter the CuNP; as a result, the SPR remained the same. At the same time, a slight aggregation was observed. The addition of troponin T resulted in a change in morphology, which resulted in a decrease in SPR along with a blue shift (Figure 6). Similarly, troponin I antibody conjugation resulted in a significant change in the size, which resulted in the broadening of the SPR. Adding troponin I resulted in an increase in the size of CuNPs affecting the SPR behavior.

The selectivities of these antibody-conjugated sensors were tested against common analytes hindering measurement such as glucose, glutamate, glycine, sodium, calcium, sucrose, and cysteine, and they were found to be highly selective for troponin T and I antigen.

## 4. Discussion

Surface plasmon resonance is one of the unique properties offered by metal nanoparticles. It depends on the nature of the material size, shape, and morphology of the nanoparticles [41,42,43]. Gold and silver metal nanomaterials of different size, shape, and morphology have been widely explored for sensing applications by utilizing the surface plasmon resonance property [44,45]. Similar to gold and silver, copper also exhibits surface plasmon properties in the visible NIR region [46,47]. Due to the inherent surface plasmon resonance of copper, nanomaterials are advantageous for plasmonic applications such as sensing and bioimaging [48,49,50,51]. The surface plasmon resonance in the near infra-red region is useful while using body fluids such as blood to avoid autofluorescence [52].

The satellite peak around 942.5 eV in the higher-resolution XPS spectra indicates the 2+ oxidation state of the CuNP. Furthermore, a transparent layer is covered by a group of 4 nm sized copper nanoparticles. The vibrational bands around 440 cm^−1^, 621 cm^−1^ and 873 cm^−1^ can be assigned to be the characteristic CuO vibrations. Similarly, a sharp vibration around 621 cm^−1^ confirms Cu-O bond formation in the CuNP nanoparticles [38,39,40]. From all these observations, we propose that the CuNP comprises a few copper clusters protected with a copper oxide layer.

Troponin T and I monoclonal antibody conjugation resulted in a change in the surface plasmon resonance of CuNP, and this can be substantiated with the help of TEM. Troponin T monoclonal antibody conjugation resulted in a slight change in the pattern of CuNP (Figure 3a). This slight change resulted in the blue shift of SPR, as shown in Figure 4a. But troponin I conjugation resulted in bigger-sized CuNPs compared to the synthesized CuNPs, which resulted in the broadening of SPR peak. Troponin T and troponin I antibody-conjugated CuNPs show concentration-dependent changes in their surface plasmon resonance properties upon adding known concentrations of troponin I and troponin T. These changes in the surface plasmon resonance properties are used for the detection of the troponin level. From TEM, it is evident that the developed nanosensor shows size and morphological changes upon adding the analytes that, in turn, affect the surface plasmon property. Such sensors made of gold nanomaterials were already used for the detection of various analytes [7,8,9,10,12]. This study mainly offers a qualitative approach for determining the efficacy of the sensor towards cardiac troponin and its mechanistic action, but a quantitative approach is more important for the detection of the troponin level in real samples. Biosensor testing on blood serum/whole blood often faces challenges such as high levels of background, crossreactivity, and baseline drift [53,54].

## 5. Conclusions

In summary, a new NIR-absorbing copper was developed for the selective detection of the cardiac biomarkers troponin T and troponin I. The synthesized nanosensor shows absorbance at around 800 nm, which is advantageous in the biomedical field. Antibodies specific for troponin T and I were conjugated with the synthesized CuNPs. Troponin T and troponin I are bonded and functionalized quickly by selective signals of the device. Its strong optical absorption based on its dielectric nature increases its efficacy as a laboratory testing device for troponin. The advantage of using the sensor is fast detection. To conclude, a cost-effective copper nanosensor was demonstrated for the selective detection of troponin T and troponin I. The goal of our future study is to check the efficacy of CuNPs toward experimental clinical samples and develop a portable point-of-care diagnostic device for the detection of both biomolecule markers: troponin T and troponin I. The potential prospect of our future study is to develop a wearable smart device that tracks troponin levels in real time, though more research would need to be conducted not only to see how this could be implemented in a non-invasive way in the human body but also for creating a sensor to analyze the blood in real time. The benefits of such a device could be early warning of cardiovascular disease and alerting the user to possible silent heart attacks, both of which could possibly save lives.

## Data Availability

The original contributions presented in this study are included in the article.
